# A protocol for systematic review and meta-analysis of the effect of acupoint therapy for essential hypertension

**DOI:** 10.1097/MD.0000000000022399

**Published:** 2020-10-02

**Authors:** Xu-dong Zhang, Ji-ping Zhao, Jiao-juan Wu, Qi Xie, Yu Wang, Qing-guo Liu

**Affiliations:** a The First Clinical College; b Department of Teaching, School of Acupuncture and Moxibustion and Tuina, Beijing University of Chinese Medicine, Beijing, China.

**Keywords:** acupoint therapy, acupuncture, essential hypertension, protocol, systematic review and meta-analysis

## Abstract

**Background:**

Essential hypertension remains an enormous public health concern, imposing a major burden of morbidity and mortality worldwide. Relevant studies showed that acupuncture therapy might be effective in treating essential hypertension. However, there is no consistent conclusion so far. The aim of this study was to assess the efficacy and safety of acupuncture therapy for patients with essential hypertension.

**Methods:**

We searched the PubMed, Embase, the Cochrane Library, Web of Science, the Chinese National Knowledge Infrastructure (CNKI), and the Wan-fang databases from inception through November 29, 2019. Randomized controlled trials investigating acupuncture therapy for hypertension were included. We will use Endnote software X8 for studies selection, Review Manager software 5.3 for the data analysis.

**Results:**

We will synthesize current studies to evaluate the safeties and effectiveness of acupuncture for essential hypertension.

**Conclusions:**

Our study will provide the evidence of acupuncture therapy for essential hypertension.

## 1 Introduction

Essential hypertension (EH) remains an enormous public health concern, imposing a major burden of morbidity and mortality worldwide.^[1]^ It is reported that 58.3% of deaths from hemorrhagic strokes and 54.5% of deaths from ischemic heart disease could be attributed to hypertension.^[2]^ It is estimated that the world's prevalence of hypertension will increase from 26.4% in 2000 to 29.2% by 2025.^[3]^ Serious consumption of medical and social resources leads to a heavy burden for families and the society, and has become an important public health issue, especially in developing countries.[^4^,^5^] In China, hypertension prevalence is rising as the population grows older and it has become the first risk factor for cardiovascular diseases burden.[^6^,^7^]

Blood pressure can be lowered by several classes of drugs and by lifestyle changes such as weight loss, salt intake restriction, and exercises. However, due to various side effects or safety concerns, such as drug resistance which could affect therapeutic efficacy, this therapy is far from satisfactory. In addition, lifestyle interventions are difficult to achieve and even more difficult to maintain. Thus, seeking for an effective and less side-effect treatment becomes an important goal for treating EH.

Acupuncture is one of the most common complementary and alternative medical therapies, has existed for 2500 years,^[8]^ which has been reported to have potential for treating EH.[^9^,^10^] Several systematic reviews have evaluated the efficiency of acupuncture for hypertension.[^11^,^12^,^13^,^14^,^15^] Recently, some new trials have been published, leading us to conduct a systematic review and meta-analysis of all available randomized for controlled trials (RCTs), to evaluate the efficiency and safety of acupuncture therapy for treating essential hypertension.

## 2 Methods

### 2.1 Study registration

This study has been registered in advance on the website of Open Science Framework (OSF, https://osf.io/) with a registration number of DOI:10.17605/OSF.IO/8R5W7. The protocol is conducted strictly based on the Preferred Reporting Items for Systematic Reviews and Meta-Analyses Protocols (PRISMA-P) guidelines. We will describe the changes in our full review if needed.

### 2.2 Eligibility criteria

#### 2.2.1 Type of study

The randomized controlled trials (RCTs) of acupuncture therapy for essential hypertension will be included. Quasi-randomized, comments, case reports, technical reports, animal studies, self-control studies, or non-RCTs will be excluded. There is no language restriction on studies selection.

#### 2.2.2 Type of participant

Based on the International Society of Hypertension Guidelines for the Management of Hypertension (1999 World Health Organization),^[16]^ essential hypertensive patients were those with a systolic blood pressure (SBP) ≥ 140 mm Hg and/or a diastolic blood pressure (DBP)  ≥ 90 mm Hg. All patients with secondary hypertension caused by an identifiable underlying primary cause were all excluded.

#### 2.2.3 Type of intervention

Acupuncture therapy included acupuncture or twirling reinforcing reducing manipulation without lifestyle modifications or anti-hypertensive drugs. Control groups received sham acupuncture or anti-hypertensive drugs or no treatment without any other treatment.

#### 2.2.4 Types of outcome measurements

Blood pressure changes are recognized to represent the effect of lowering blood pressure.

Primary outcomes included SBP and DBP changes (post-treatment BP—pre-treatment BP). Secondary outcomes included the adverse events.

### 2.3 Search strategy

We systematically searched the PubMed, Embase, the Cochrane Library, Web of Science, the Chinese National Knowledge Infrastructure (CNKI), and the Wan-fang databases for inclusion on November 29, 2019 with MeSH terms and key words, and without language restrictions. Search strategy terms were (acupuncture OR acupoint OR twirling reinforcing reducing manipulation) AND (high blood pressure OR hypertension OR essential hypertension) AND (randomized controlled trial OR controlled clinical trial OR randomized OR clinical trials). We will also scan the relevant published references carefully to identify further publications. When there are questions related to the results of the study or trial design, corresponding authors will be contacted to confirm the information that we extract from their studies or to eliminate any ambiguity.

### 2.4 Data collection and analysis

#### 2.4.1 Study selection

**Figure 1:**
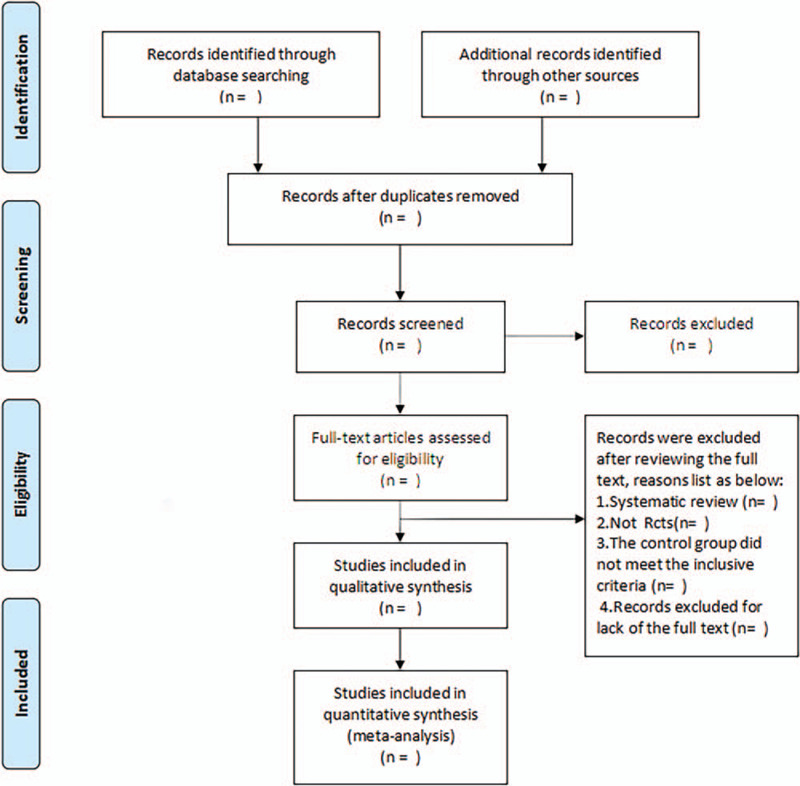
Flowchart of literature selection.

All articles retrieved will be imported into endnoteX8 to remove the duplication studies. The two authors (XDZ and JPZ) will independently scan the title and the abstract of every record to exclude irrelevant articles. The full text of the qualified articles will be investigated and then the authors will select articles that meet the inclusion criteria. Disagreements were resolved in consultation with the third reviewer (QGL). The research flowchart is shown in Fig. 1.

#### 2.4.2 Data extraction and management

Two reviewers (XDZ and JPZ) screened all the literature and extracted data independently using a standardized form. The form was pre-designed for collecting information on trial characteristics, including first author, language, number of patients, mean age of the patients, diagnostic criteria, grades of hypertension, acupuncture treatment, control types, sessions of treatment, treatment course, outcome measures, and adverse. We defined the change values of blood pressure as post-treatment BP minus pre-treatment BP and extracted the change means and standard deviations (SD) for continuous outcome. For dichotomous outcome measures, we used rates (the number of events out of total number in the study). If change means and standard deviations were missing, we calculated them according to the formula offered by the Cochrane Handbook for Systematic Reviews of Interventions (Version 5.3). Disagreements were resolved in consultation with the third reviewer (QGL).

#### 2.4.3 Assessment of risk of bias in included studies

Two reviewers (QGL and JJW) assessed the risk of bias of the included RCTs using the Cochrane Collaboration's tool for assessing risk of bias. Each trial was scored as high, low, or unclear risk for the following seven domains: (1) random sequence generation (selection bias); (2) allocation concealment (selection bias); (3) blinding of participants and personnel (performance bias); (4) blinding of outcome assessment (detection bias); (5) incomplete outcome data (attrition bias); (6) selective reporting (reporting bias); (7) any other bias. Disagreements were resolved in consultation with the third reviewer (XDZ).

#### 2.4.4 Statistical analysis

Statistical analysis will be performed by using Cochrane Review Manager (RevMan 5.3) software when a meta-analysis is allowed. Dichotomous data represents the risk ration (RR), and continuous data represents the mean deference (MD) when the outcomes are measured in the same way among different trials. 95% of the confidence interval (CI) will be used as an effective size for the combined analysis.

#### 2.4.5 Assessment of heterogeneity

Statistical heterogeneity across trials was assessed by the Cochran Q test (*P* < 0.1 for statistical significance) and quantified by the *I* ^2^ statistic. Following the Cochrane Handbook for Systematic Reviews of Interventions (Version5.3), we defined *I* ^2^ > 50% as indicating significant heterogeneity. Heterogeneous data were pooled using the random-effects model.

#### 2.4.6 Analysis of subgroups

We performed subgroup analysis based on the classes of anti-hypertensive drugs such as calcium channel blockers (CCB), β-receptor antagonists, angiotensin-converting enzyme inhibitors (ACEI), and angiotensin receptor blockers (ARB). Meta-analysis was performed using RevMan 5.3 software.

#### 2.4.7 Sensitivity analysis

When sufficient data are available, sensitivity analysis will be performed to test the robustness of the primary outcomes, which includes assessing the quality of the methods, the quality of the studies, and the impact of sample size and missing data.

#### 2.4.8 Assessment of reporting biases

The results of the meta-analysis will be presented in the form of a forest. If the studies included in meta-analysis are more than 10, funnel plot will be used to evaluate potential publication bias.

#### 2.4.9 Confidence in cumulative evidence

The level of evidence on outcomes will be assessed utilizing the Grading of Recommendations Assessment, Development and Evaluation (GRADE).^[17]^ Based on this grading systems, the result will be categorized as high, moderate, low, and very low quality.

### 2.5 Ethics and dissemination

Since our study have no connection with individual patient data, thus, it is not necessary for ethical approval. The results of our systematic review will be reported on a peer-reviewed journal or relevant conferences to provide the implication of acupuncture therapy for patients with EH.

## 3 Discussion

Hypertension is an increasingly important medical and public health issue, and the benefits of lowering BP, such as reductions in the incidence of stroke (average reduction, 35%–40%), myocardial infarction (20%–25%), and heart failure (>50%), are significant. Blood pressure can be lowered by several classes of drugs and by lifestyle changes such as weight loss, salt intake restriction, and exercises. However, due to various side effects or safety concerns, such as drug resistance which could affect therapeutic efficacy, this therapy is far from satisfactory. Acupuncture has long been a therapeutic modality in East Asia, and is being increasingly accepted in the west, which has been reported to have potential for treating EH. Several systematic reviews have been performed to evaluate the efficacy and safety of acupuncture for essential hypertension. However, the current systematic review updates the latest evidence, focus on the twirling reinforcing-reducing manipulation for the first time. Twirling is the technique of acupuncture, as to achieve the purpose of strengthening the stimulation. It is also one of the common methods of the Chinese acupuncture; therefore, it is similar to the regular needling. Due to the limited number of relevant high-quality studies and the few sample sizes included, the strength of the arguments of the conclusions is to some degree limited. Therefore, we hope that more large-scale, high-quality RCT should be necessary in the future.

## Author contributions

**Data curation:** Xu-dong Zhang, Ji-ping Zhao.

**Formal analysis:** Jiao-juan Wu, Qing-guo Liu.

**Project administration:** Ji-ping Zhao, Qing-guo Liu.

**Resources:** Xu-dong Zhang, QI Xie.

**Software:** QI Xie, Jiao-juan Wu.

**Supervision:** Ji-ping Zhao, Yu Wang.

**Writing – original draft:** Xu-dong Zhang.
